# The Pathology of Involuntary Spermatic Fluxes

**Published:** 1881-09

**Authors:** George H. Picard

**Affiliations:** Topeka, Kan.


### ARTICLE IV.

THE PATHOLOGY OF INVOLUNTARY SPERMATIC FLUXES. A Paper read before the Eastern Kansas Medical Society. By GEO. H. PICARD, M.D., Topeka, Kan.

The subject I have chosen for this paper has by no means been exhaustively treated by American medical talent. Indeed, the prudent surgical *litterateur* chooses rather to pass it by, so malodorous has it become in the hands of charlatans and self-constituted "sexual pathologists." Excepting the admirable monograph of our distinguished countryman, Roberts Bartholow, and an English translation of Lallemand's "*Des pertes Seminales Involuntaires*," I know of no work in our language which is exclusively devoted to the subject, which now-a-days seems to be regarded by the surgeon with much the same distrust as the careful husbandman exhibits toward the well directed approaches of the fragrant quadruped scientifically known as *mephitis chiuga.*

For much the same reason, I opine, nothing is more evident than the fact that the general practitioner of medicine and surgery knows very little either of the pathology or treatment of spermatorrhœa; not, indeed, because there is any especial difficulty connected with the proper investigation of the subject—I mean in the learning of whatever is to be taught about it—but rather because the merit to be gained in this direction is so inconsiderable that most men prefer to labor in a field less approximate to quackery and more certain of repute.

As is often the case, the first difficulty in the discussion of spermatorrhœa is to be found in the definition. What is spermatorrhœa? Is it, as the term seems to imply, a constant stillicidium of semen from the external urethra? Or, does it embrace all discharges except those of a known specific origin, which may at any time escape from the urethral orifice? Is it a disease? Is it a symptom? Is ti a neurosis? Is it a local lesion? What is it? To say that it consists in a mere passive stillicidium of semen without the accompanying phenomena of erection and ejaculation would include a collection of instances so rare as to almost do away with the necessity for a definition at all, and very likely exclude a far greater number of cases entirely pathological in character. On the other hand, it is not safe to affirm that every seminal-like discharge issuing from the urethra is actually seminal, for it is altogether likely that most of these fluxes do not contain spermatozoa, and are the result of irritation and hyper-secretion of the mucous surfaces of the canal.

At this point, lest it should appear that I am trying to shirk the responsibility of defining spermatorrhœa, I will give my notion of it, and although I am aware that it is by no means exact, I am persuaded that it includes nearly everything essential to a definition. *Spermatorrhœa embraces all involuntary spermatic fluxes occurring too frequently to be regarded as physiological.*

About fifty years ago M. Lallemand, the distinguished surgeon of Montpellier, expended much care and research in the investigation of the pathology of this disorder. So clever, so lucid, and withal so dogmatic were the results of this scholarly search as given to the world in the work, published in 1837, entitled "*Des Pertes Seminales Involuntaires,*" that the author's views were almost universally adopted wherever surgery was known. Indeed, at the present time, most English and many continental surgeons cling fondly to the dicta of the surgeon of Montpellier, and I predict a revival of his doctrines in this enlightened country.

In order to make my observations clear, it will be necessary to preface them with a brief *résumé* of Lallemand's theory. The central idea in this surgeon's theory of the pathology of spermatorrhœa is the production, by various causes, of an irritation of the prostatic portion of the urethra and the seminal ducts. The following causes play an important *rôle* in the instigation of the disorder : Blenorrhagia and stricture of the urethra ; gouty and rheumatic affections of those parts ; sebaceous accumulations beneath the prepuce ; venereal excesses and masturbation ; excessive use of diuretics, cantharides, ergot, etc.; the abuse of alcoholic stimulants, and of tea and coffee ; constipation ascarides, hæmorrhoids, etc.

M. Lallemand divides seminal losses into *diurnal* and *nocturnal.* Diurnal fluxes, according to the observer, are of vastly less frequent occurrence than those of a nocturnal character, although they are quite as serious, if, indeed, not more rebellious to treatment. They take place, for the most part, passively, without erection or ejaculation, during or just after defecation and micturition. It is not common to find patients suffering from both daily and nightly fluxes. The nocturnal losses are frequently connected with all the phenomena of sexual excitation, becoming pathological by reason of their involuntary nature and their abnormal frequency.

M. Lallemand found the results of genuine and well established spermatorrhœa to be speedily disastrous, the mischief being due to local injuries and important functional disturbances. "The penis becomes relaxed, the erections feeble; the *corpora cavernosa* either atrophy or their vessels lose tonicity, the *corpus spongiosum* and the *glans penis* also shrink; the testes undergo a certain degree of atrophy; the superficial veins of the penis become dilated and tortuous, etc. The subjective symptoms begin after a longer or shorter time to be marked: pains in the lumbar region; aching in the arms and testicles; the appetite is capricious, the digestion feeble; the bowels become deranged, constipation alternating with diarrhœa. The nervous system very often manifests sympathetic disturbance in the forms of vertigo, pains along the courses of the principal nerves, etc.

As the outcome of his microscopical research, M. Lallemand teaches as follows: "When the spermatorrhœa has assumed sufficient importance to influence the rest of the economy, the semen becomes more liquid and the spermatozoa are less developed and less vivacious, although their number may not appear to be diminished. When the erections begin to diminish, the semen is still more aqueous, the dimensions of the spermatozoa are sometimes one-fourth their normal size, their caudal prolongation being hardly distinguishable with a power of 300 diameters." In support of his theory of the local nature of spermatorrhœa, M. Lallemand brings to bear a far greater amount of cadaveric observation than any of his successors, be they friends or foes, and in a supplemental treatise, published in 1839, he stoutly maintains the correctness of his early views.

Opposed to Lallemand and the Montpellier school are the great clinician Trousseau, in France, the neurologist Rosenthal, in Germany, and our not less distinguished countryman, Roberts Bartholow. Surgeons, as a rule, adhere to the teachings of M. Lallemand, thus resisting the encroachments of the all absorbing neurologists.

In giving a short account of the neuro-spermatists, as I propose, for the sake of convenience, to call all those who believe spermatorrhœa to be a actual neurosis, I shall avail myself of the fascinating monograph of Prof. Bartholow, which has lately reached its fourth edition, an event in medical literature in itself worth recording. "Spermatorrhœa," says Prof. Bartholow, not a whit less dogmatically than Lallemand, "is a neurosis. Although structural alterations may be coincident, they are not causative." That the disorder is a local lesion, Bartholow claims is unsupported by the evidence of morbid anatomy, and he does not hesitate to insinuate that Lallemand's cases were selected and his theory constructed to justify his practice. In support of his opinion, he first cites the authority of Mr. Thompson, who asserts that "sexual indulgence cannot have the effect of producing prostatitis, unless gonorrhœa be already existing." He further places in opposition to the views of M. Lallemand his own personal observations. It appears from his account, that he has lately made the dissection of a young man, dead of a double pneumonia, who was known to have practiced masturbation for many years, (whether or not this young man had spermatorrhœa he does not state). He informs us that he found no lesion beyond a catarrhal condition of the seminal and prostatic ducts and of the *vesiculæ seminales.* On this ground he declares the position of Lallemand untenable. He also maintains that the views of Lallemand in regard to the alterations in the spermatozoids are hardly less fanciful than his ideas on local structural alteration. In confirmation of this, he uses the experience of Liégeois, who out of six cases under observation, claims to have found only one case of azoospermia. Bartholow objects very positively to Lallemand's division of *diurnal* and *nocturnal.* He regards the existence of passive diurnal emissions to be purely mythical, and explains that the passive discharges which sometimes occur during and after defecation and micturition, are not seminal in nature, being entirely destitute of spermatozoa. In this view he claims the support of Austin Flint* and many other original observers.

In spite of the literary and speculative fascination attached to these recent theories, I am, from a rational standpoint, a firm believer in the correctness of Lallemand's teachings. It seems to me that exact neurology is already sufficiently comprehensive without spermatorrhœa. It is beginning to appear that whatever the ambitious gynæcologist has not already included in his specialty is about to be appropriated by the equally enterprising neurologist. Thus is the once fair domain of the general practitioner being parcelled out among the specialists. That the pathology of spermatorrhœa consists in structural changes, manifested locally in the generative apparatus, appears rational to me for very many reasons.

Prof. Bartholow's charge against Lallemand's sincerity in the matter of the selection of his cases for cadaveric observations seems to me to be trivial and unworthy of Bartholow's great reputation. Lallemand did not base his opinion on a single observation, as Bartholow seems to have done; on the contrary, he deducted his theory of structural change from numerous carefully conducted observatious extending through a period of over fifteen years. It is also a significant fact that subsequent observers have confirmed M. Lallemand's assertions, *e.g.*, M. Raige. De Lorme, in his "Dictionary of Medicine," states that "in cases of spermatorrhœa, diverse alterations of the genital organs have been found," and, indeed, the fact has never been disputed until Prof. Bartholow *did not* find structural alteration *in his one case;* and it cannot but be evident that for the value of the evidence it would have been better to have stated whether or no the man had spermatorrhœa.

Prof. Bartholow has no faith in the phenomena of passive

* What Flint really says is this: "In the most of these cases the fluid is either the *liquor prostaticus* or a secretion from the *vesiculæ seminales.* The microscope affords the only means of determining that the fluid is seminal."

diurnal spermatorrhœa. He considers the fluxes which occur during and after micturition and defecation to be mostly of a mucous character, and less frequently the pathological relief of certain surcharged and plethoric individuals whose secretory functions are greater than their retentive capacities. In support of this belief, he quotes Dr. Chambers, of St. Mary's Hospital, who states that this discharge "has no more pathological significance than the leucorrhœa of women,"—whatever that may mean. To assert that the leucorrhœa of women is devoid of significance is to fly in the face of modern gynæcology. With a somewhat limited experience—I have a record of five cases of this kind—I am prepared to substantiate the correctness of M. Lallemand's views on this point. It may not prove wholly uninteresting if I relate the history of a case, which, at the time, I regarded as especially illustrative of the matter in question: An American of robust appearance, aged thirty-three years; married seven years; father of one child five years of age; by his own confession, inordinately addicted to venery. About three years ago, he first became conscious that a passive urethral flux followed defecation at intervals of three or four days; suffered no diminution of his sexual appetite for two years, but thinks that for the past year his desires are less importunate; no falling off in weight; no symptoms of nervous disorder; no nocturnal emissions; has experimented as to the effects of living *absque marito*, and finds that the passive fluxes continue and that they are not superseded by nocturnal ones. I have repeatedly examined these fluxes with the microscope and have always found them to be seminal. At this point I may remark, that I have more than once had my faith shaken in regard to the soundness of Prof. Bartholow's microscopic efforts. He confesses that even in known cases of spermatorrhœa which had existed for years, he has never found any change in the number or configuration of the spermatozoa. For my part, I can very decidedly affirm that I have seldom found the spermatozoids unaltered, the most noticeable change being a shortening of the caudal filament. I have not observed that there is a marked diminution in numbers; but that there is a positive decrease of motion, and that the caudal prolongations of the spermatozoids are very considerably reduced, is clearly apparent with a microscope of only 300 diameters. I have repeatedly verified this fact and am quite satisfied that it will stand the test of criticism. It cannot be denied that there is a very close connection between the local phenomena of spermatorrhœa and a series of neurotic symptoms which in a large number of cases are likely to supervene. It is equally true that all other local lesions may be accompanied by the self-same nervous disturbances, always bearing in mind the fact, that lesions of the reproductive system—for reasons which are obvious—are less heroically borne than injuries in any other part of the human anatomy. No one can have failed to observe that all chronic disorders are often productive of melancholia and extreme nervous prostration. I have seen as great mental depression arising from the existence of an incurable ulcer on the leg as could be possible even in the most advanced stages of spermatorrhœa. The whole structure of modern gynæcology rests upon the neurotic sequences induced by primary uterine lesions.

It seems to me that the theory of the exclusively neurotic character of spermatorrhœa proves too much. That the nervous system exercises a peculiar influence over the glandular apparatus is undoubtedly true. Is any neurosis causative of prostatorrhœa or blenorrhagia? Review for a moment the morbid anatomy of spermatorrhœa (according to the Montpellier school, which I prefer by reason of its greater accuracy): There are ulcerations of the orifices of the ejaculatory ducts; injection and ulceration of various portions of these canals; similar alterations in the *vesiculæ seminales;* purulent deposits in the vesicles, in the *vasa efferentia*, in the epidermis, in the body of Highmore, and in the testicle. Now, in prostatorrhœa there is precisely an analogous condition of the ducts; yet, as far as I know, no neurologist has proclaimed the sovereignty of the spinal cord over this disorder. Is not the abnormal increase of function in one series of glandular apparatus as easily to be accounted for as in another?

Bartholow teaches that genuine spermatorrhœa is exceedingly rare, and that most cases that present themselves for treatment are purely imaginary. I believe this to be fortunately true, but does it not somewhat militate against the neurotic theory? If spermatorrhœa be purely nervous in its origin, would it not, in view of the enormous existing causative influences, be an every day matter? In these degenerate days of shattered nerve forces, habitual excesses and social corruption, one might indeed shudder at the unwholesome possibilities suggested by the neurotic idea! I grant that this theory is excellently well adapted to imaginary spermatorrhœa, but for the pathology of true spermatorrhœa I prefer something more rational.

But the most telling testimony against the theory of Trousseau and Bartholow may be found in its therapeutical sequel. I am not unwilling to admit that anaphrodisiacs may be useful in a placeboic sense, and that nerve-stimulants—so called—may serve to buoy up the despairing fortunes of the hesitating sexual neophyte; but I do not believe that a case of real spermatorrhœa has ever been cured by any one or by all of them. Clinical medicine must have shown this to be true.

A fact worthy to be noticed is, that in spite of the strictly neurotic ideas enjoined by both Trousseau and Bartholow, they rely very greatly upon local and mechanical treatment. M. Trousseau has even achieved his most satisfactory results from the employment of local empiricism, such as the forcible dilatation of the *sphincter ani*, and the insertion of a boxwood block into the rectum. Bartholow does not hesitate to avow his lack of confidence in nerve-stimulation and brain-food; he seems to find his most reliable agent in hydropathy, applied in the form of the continuous cold current. I have not observed that he explains the therapeutics of the continuous aqueous current in any original or strikingly neuropathic manner.

If an account of the rational treatment of spermatorrhœa were demanded of me, I should say: Be certain of the diagnosis,—it is a somewhat rare disorder, and all passive fluxes from the urethra should be doubtful until the microscope has told the story; treat nervous complications as intelligently as you would those arising from any other cause; and use some approved form of local treatment. I agree with Bartholow, that the *porte-caustique* of Lallemand is a somewhat dangerous agent in unskillful hands, but it is very efficient in the hands of the surgeon who knows the parts and the instrument. The tanno-glycerine is about as desirable as the caustic and has the merit of being harmless. I have injected a dram or more of the tanno-glycerine—ʒ–℥—at intervals of a week or so until a cure was effected. These injections may be made in various ways. The universal syringe can be attached by means of the screw to a silver catheter, which can be introduced into the prostatic urethra. There are hard-rubber syringes especially constructed with long, curved, bulb-tipped nozzles, which answer the purpose most admirably. Various other astringent injections are of great service, the only requisite on the part of the surgeon being ordinary skill and attention.

